# Construction of a Medical Resource Sharing Mechanism Based on Blockchain Technology: Evidence from the Medical Resource Imbalance of China

**DOI:** 10.3390/healthcare9010052

**Published:** 2021-01-06

**Authors:** Hu Liu, Yuxuan Liu

**Affiliations:** International Business School, Shaanxi Normal University, Xi’an 710119, China; liuyuxuan@snnu.edu.cn

**Keywords:** blockchain, medical resources, mechanism of sharing

## Abstract

Health equity is a very important part of social equity. The outbreak of the novel coronavirus pneumonia (COVID-19) in a short period of time exposed the problems existing in the allocation of medical resources and the response to major public health emergencies in China. By using Kernel density estimation and Data envelopment analysis (DEA), it is found that the allocation and imbalance of medical resources in China are greatly different among regions, and the polarization phenomenon is obvious. As an important part of the information technology system, blockchain technology is characterized by decentralization and non-tampering. It can realize sharing of medical resources through a mechanism of resource storage, circulation, supervision, and protection. The construction of a medical resource sharing mechanism under the condition of blockchain technology will greatly improve the degree of medical resource sharing, will narrow the differences in resource allocation between regions, and can effectively respond to an outbreak of major public health emergencies.

## 1. Introduction

In December 2019, the COVID-19 outbreak began on the eve of the New Year and spread rapidly around the world. In January 2020, the World Health Organization declared COVID-19 to be an international public health emergency. Since then, China has entered a period of nationwide anti-epidemic efforts. In his speech at the National COVID-19 Recognition Conference, general secretary Xi Jinping mentioned that the COVID-19 epidemic is the most serious infectious disease pandemic in the world in the past century and that it is the major public health emergency with the fastest spread, the widest range of infection, and the most difficult prevention and control since the founding of the People’s Republic of China [1,2,3]. In order to effectively reduce social risks and to control the spread of COVID-19, The State Council quickly launched a joint prevention and control mechanism and mobilized high-quality medical resources from all over the country to Hubei province to effectively respond to the outbreak of COVID-19. During this period, China’s response to the transport of a large number of medical aid resources was coordinated through a small number of organizations, taking the Red Cross as an example. However, when a public health emergency like COVID-19 suddenly occurs, a large number of medical supplies and life supplies flood in at the same time, and traditional resource management mechanism and resource operation channels are unable to cope with the large number of resources gathering in a short time, thus causing a phenomenon called “crash”. As a result, many emergency medical supplies are overstocked in warehouses, unable to reach the front line to fight against the epidemic. This exposes the contradiction between the supply and demand of medical resources in China and the incoordination of the supply chain of medical resources in responding to major public health emergencies.

Since Satoshi Nakamoto published the white paper on Bitcoin [4] and the first Bitcoin came into being in 2009, the blockchain, as the supporting technology of Bitcoin, has gradually come into people’s sight and has developed rapidly. The year 2015 was known as the first year of blockchain, and search records with blockchain technology as a key word continues to top the list. Since then, blockchain has ushered in a spurt of development, attracting extensive attention from the industry and academia. According to the existing literature, the academic community has not yet formed a unified definition or concept of blockchain. Some scholars regard blockchain as a database with decentralized characteristics [5,6], while some scholars define blockchain as distributed ledger technology [7,8]. The characteristics of blockchain, such as decentralization, de-trust, transparency, and non-tampering, have aroused discussions among experts and scholars in different fields. Blockchain not only can influence existing businesses but also can provide the possibility for new business directions through the application of blockchain technology. Blockchain is a new platform technology which complements the lack of privacy, reliability, and scalability of emerging technologies. For example, the results obtained by Christidis and Devetsikiotis (2016) show the potential of intelligent contracts to securely support transactions between devices in the Internet of Things environment [9]. On this basis, Korpela (2017) [10] found through research that transaction processing and the timestamp of blockchain technology can usually be used for supply chain management. At present, research on the impact of blockchain technology on commercial value has attracted more and more attention. Through a combination with Internet of Things services, many researchers believe that blockchain technology has great transformational abilities in multiple industries. Researchers mainly studied the markets and industries that will be affected by blockchain. Financial markets [11,12], logistics, public services [13], and other fields also received much attention. In addition to these fields, researchers have also studied the business value of blockchain in various industries, such as the transportation [14,15], power market [16], and electronic health sectors [17].

After the outbreak of COVID-19, uneven medical and health resource allocation and health emergency system failure received widespread attention from all walks of life. However, in fact, since the 21st century, academic research on the differences in medical and health resource allocation has been relatively mature [18,19,20,21], with the existing research mainly based on two aspects: population distribution and geographical distribution of medical resource allocation fairness. Many researchers believe that the fairness in medical and health resources according to population distribution is significantly better than that according to geographical distribution in some regions of China and that the difference between regions is an important factor affecting the fairness of health resource allocation [22,23]. The reasonable and optimal allocation of medical and health resources is of great importance to the development of China’s health industry [24]. Some scholars have also studied the relationship between application of the Internet and personal medical satisfaction and life satisfaction [25,26], and the impact of Internet applications on environmental quality [27] and environmental protection [28], explaining to a large extent the penetration of Internet applications into every aspect of life. However, development of the Internet and information technology has yet to be fully applied in breaking the barriers of regional resource differences and in constructing a sharing mechanism for medical resources.

As early as 2010, information system research proposed the digitization of medical systems based on the application of information technology in the medical field at that time. Then, the research results of the application of an information system in the medical field were summarized and the prospect of developing medical and health causes driven by technology was made. At present, the application research on blockchain in the medical field is mostly based on the decentralized, untamperable characteristics of blockchain technology itself. In recent years, researchers have been committed to building an electronic medical record management system by using blockchain technology, such as MedRec [29], MedShare [30], and Medblock [31]. In addition, Dubovitskaya A (2018) [32] proposed a framework for managing and sharing electronic medical records data for cancer patient care based on blockchain medical data management. Li H (2018) [33] proposed a novel medical data storage system (DPS) based on blockchain. The difference is that more prudent data storage strategies and various encryption algorithms are used to ensure user privacy. Chen Y (2019) [34] designed a service framework for sharing medical records based on blockchain and cloud storage to store personal medical data, aiming to break the information island phenomenon in medical data.

Health equity is an important embodiment of social equity, and promoting the trans-regional flow of medical resources can effectively promote the development of China’s medical and health undertakings and thus promote the realization of social equity. At present, there are still some problems in China, such as unbalanced allocation of medical and health resources and failure of the health emergency system. The specific application of Internet information technology represented by blockchain in the medical field still needs to be explored. Therefore, this paper will start with the differences in the allocation of medical resources in China, will study the mechanism realizing optimal allocation of medical resources based on blockchain technology, and will explore the specific path realizing sharing of medical resources across organizations and regions.

## 2. Background: Medical Services and the Development Situation of Blockchain in China

In 2016, the Central Committee of the Communist Party of China (CPC) and The State Council made it clear that “joint contribution and shared benefits for all” is the strategic theme in building a healthy China, which is also the essential requirement for implementing one of the five development concepts of “sharing” and “development for the people, development by the people and development fruits shared by the people”. In response to the COVID-19 outbreak, medical institutions and disease prevention and control institutions in China are generally in short supply of medical supplies. When large public health emergencies occur, no city or province can handle herself in the short period of time when many patients are diagnosed with infectious phenomena, exposing the Chinese medical resource allocation imbalance and health problems such as failure of the emergency system. Thus, construction of a medical resource sharing mechanism is of necessity and urgency. Science and technology provide strong supports for elevating China as a great power. At the 18th group study session of the Political Bureau of the CPC Central Committee, Xi Jinping stressed the use of blockchain technology to promote greater connectivity in information, capital, talent, credit investigation, and other areas. In this context, blockchain technology provides technical support for the construction of a medical resource sharing mechanism. Sharing is an essential requirement of socialism for the Chinese. It is of great significance to build a blockchain-based medical resource sharing mechanism, both for balanced allocation of medical resources and for an effective response to public health emergencies.

On the basis of exploring the current situation of medical resource allocation in China, this paper analyzes the current situation of medical resource sharing in China, analyzes the coupling between blockchain technology and a medical resource sharing system, and explores the construction of a medical resource sharing mechanism under the condition of blockchain technology.

## 3. Methodology

### 3.1. Kernel Density Estimation

Kernel density estimation is an important nonparametric estimation method for the study of unbalanced distribution. It is more flexible than parametric methods to infer regression surface based on data structure without presetting the specific form of a function. Kernel density estimation is mainly used to estimate the probability density of random variables. The distribution form of random variables is represented by a continuous density curve. The changes in image position, shape, and ductility (left and right trailing) represent the changes in distribution gap size and polarization phenomenon, and the formula is as follows:

(1) $$f\left(x\right)=\frac{1}{Nh}\overset{N}{\underset{i=1}{\sum }}K\left(\frac{x_{i}-x}{h}\right)$$

In the above equation, f(*x*) is the density function of the random variable *x* and represents the probability density at point *x*; the Kernel function is expressed with *K*; $x_{i}$ is the observed values (independent, identically distributed); *x* is an average; *N* is the number of observations; *h* is the bandwidth; and the choice of bandwidth determines the smooth degree of the Kernel density estimation and estimation precision, in general, with the larger the bandwidth (small), the smoother the density function curve (not smooth) and, the fewer samples, the greater the bandwidth.

Kernel density estimation mainly includes triangulation, quadrature, Gaussian, and leaf Panicnikov types. In this paper, the form of a Gaussian Kernel function is selected, and the specific expression is shown below.

(2) $$K\left(x\right)=\frac{1}{\sqrt{2\pi }}exp\left(-\frac{x^{2}}{2}\right)$$

### 3.2. Data Envelopment Analysis Model

The purpose of a data envelopment analysis model is to evaluate the relative efficiency of each decision unit by using a mathematical programming method in the case of multiple inputs and outputs. The Data Envelopment Analysis (DEA) model does not directly synthesize data, so the optimal efficiency index of the decision-making unit is independent of the selection of the input index and output index, does not need to estimate parameters in advance and to assume the index weight, and does not need to conduct dimensionalized processing of index data. The first DEA model is known as the CCR model (abbreviated to C2R model), which is created by American scholars Abraham Chane and William Helwig cooper in 1978. The DEA model is a system analysis method based on the concept of "relative efficiency", according to the multiple indicators and indicators of output of the same type of units (departments) of relative effectiveness and efficiency evaluation [35]. On the assumption the scale reward, DEA model can calculate the overall comprehensive efficiency. Then, under the assumption of variable scale reward, the BBC model which can calculate the scale efficiency and technical efficiency of the DMU (decision making units) was created.

DEA is a basic model of the CCR model of decision-making unit for effectiveness in scale and technology and, at the same time, for effectiveness of the overall efficiency, including the input-oriented model of certain cases seeking minimum input (output) and the output-oriented model (in certain circumstances to seek maximum output). This paper chose the output-oriented model of medical resources in allocation differences.

(3) $$s.t.\{\begin{array}{c} \overset{n}{\underset{i=1}{\sum }}x_{ij}\lambda_{i}+s_{j}^{-}=\theta_{0}X_{j0}\;\;\;\;\;\\ \overset{n}{\underset{i=0}{\sum }}Y_{n}-S_{r}^{+}=Y_{r0}\;\;\;\;\;\;\;\;\;\\ \theta_{0}\geq 0,\lambda_{i}\geq 0.i=1,2,\cdots ,n\\ s_{j}^{-}\geq 0,s_{r}^{+}\geq 0.j=1,2,\cdots ,n \end{array}$$

In the model expression of CCR, $X_{i}={\left(x_{1i},\cdots ,x_{mi}\right)}^{T}$, $Y_{i}={\left(y_{1i},\cdots ,y_{mi}\right)}^{T}$, $x_{mi}$ is the *m*th input of the *i*th DMU and $y_{mi}$ is the *m*th output of the *i*th DMU. $s_{j}^{-}$ output underproduction) and $s_{r}^{+}$ input redundancy) are the introduced relaxation variables. When the resource allocation efficiency of the decision-making unit reaches the optimal level, DEA is effective and has the highest performance, $\theta =1,\;s_{j}^{-}=0,\;s_{r}^{+}=0$ at this time. If only θ=1, then the resource allocation efficiency does not reach the optimal state and the decision unit DEA has weak efficiency.

(4) $$s.t.\{\begin{array}{c} \overset{n}{\underset{i=1}{\sum }}x_{ij}\lambda_{i}+s_{j}^{-}=\theta_{0}X_{j0}\;\;\;\;\;\\ \overset{n}{\underset{i=0}{\sum }}Y_{n}-S_{r}^{+}=Y_{r0}\;\;\;\;\;\;\;\;\;\\ \theta_{0}\geq 0,\lambda_{i}\geq 0.i=1,2,\cdots ,n\\ s_{j}^{-}\geq 0,S_{r}^{+}\geq 0.j=1,2,\cdots ,n\\ \overset{n}{\underset{i=1}{\sum }}\lambda_{i}=1\;\;\;\;\;\;\;\;\;\;\;\;\;\;\; \end{array}$$

Under the assumption of variable scale reward, the BCC model as shown above can be obtained by adding a variable scale constraint. Combined with the BCC model and CCR model, the comprehensive efficiency, technical efficiency and scale efficiency of the decision-making unit can be obtained.

The index selection of DEA model is shown in Table 1. The input of resources is mainly considered from the aspects of human and material resources. Due to the lack of total value data for medical equipment in all provinces and cities, according to the statistical indicators provided by the China Health Statistics Yearbook, the selected input indicators include the number of medical institutions per 10,000 people, the number of beds per 10,000 people, and the number of health technicians per 10,000 people. Output indicators include total visits to medical institutions and total discharges from medical institutions.

In order to comprehensively analyze the allocation efficiency of medical and health resources of all provinces and regions in China and, at the same time, to consider the requirements of the DEA model on the indexes of decision-making units and data comparability, this paper chooses 31 provinces and regions in China except Hong Kong, Macao, and Taiwan as the decision-making units of the DEA model, which fully meets the minimum requirements of the DEA model on sample size. Based on the principle of rationality and the availability of data, data such as the number of medical and health technicians and the number of medical institutions are all from the 2019 National Economic and social Development Statistical Bulletin and the 2010–2018 China Statistical Yearbook of provinces and cities, and the relevant data of medical institutions are from the official website of the National Health Commission.

## 4. Results

### 4.1. Kernel Density Estimation

**Figure 1:**
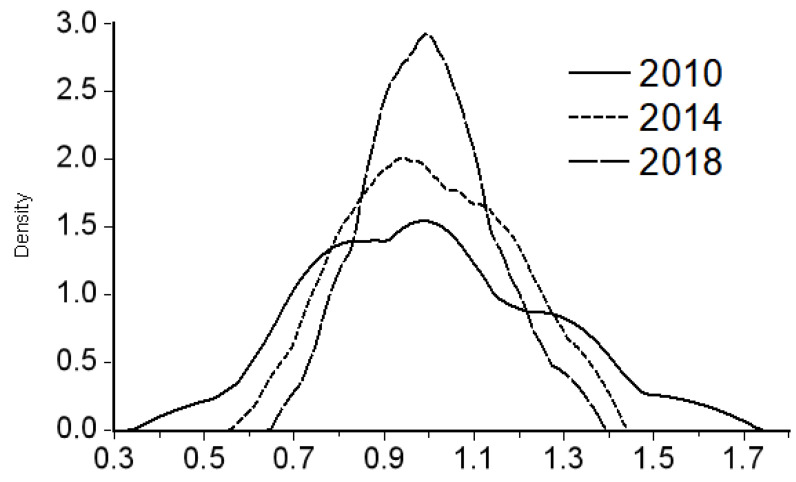
Kernel density estimation of the number of health personnel per 10,000 people in western economic belt provinces.

In order to explain the dynamic changes in the differences in the allocation of medical resources, this paper uses the data of health personnel per 10,000 people from 2010 to 2018 to estimate the Kernel density curve of the three economic zones in the west, the middle, and the east through Eviews7 software and selects 2010, 2014, and 2018 as the study years. In Figure 1, the vertical axis represents the corresponding Kernel density value, which reflects the relative change of medical resource allocation, and the horizontal axis represents the multiple of the population mean. A value of 1 represents that the number of health personnel per 10,000 people in the sample economic belt province is equal to the overall average level (see Figure 1). Figure 1 Shows the Kernel density estimation of health personnel per 10,000 people in the western economic belt (Sichuan, Guizhou, Yunnan, Chongqing, Tibet Autonomous Region, Shaanxi, Gansu, Qinghai, Ningxia Hui Autonomous Region, Xinjiang Uygur Autonomous Region), the central economic belt (Beijing, Tianjin, Shanxi, Hebei, Inner Mongolia Autonomous Region, Henan, Hubei, Hunan, Guangdong, Guangxi Zhuang Autonomous Region, Hainan) and the eastern economic belt (Heilongjiang, Jilin, Liaoning, Shanghai, Jiangsu, Zhejiang, Anhui, Fujian, Jiangxi, Shandong), from left to right.

The distribution dynamic diagram of provinces in the western economic belt (see Figure 1) shows the following characteristics: First, during the sample investigation period, the Kernel density curve as a whole does not show a trend of left and right movement, indicating that the overall medical resource investment level of provinces in the central economic belt is stable. Second, the Kernel density curve is shortened and, at the same time, peak height is increased and peak width is decreased, which reflects that the overall allocation difference of medical resources is reduced and the range difference shows a trend of continuous decrease. Thirdly, from 2010 to 2018, the Kernel density curve evolved from a weak two-peak trend into a clear single-peak pattern, indicating that the allocation of medical resources in western economic belt provinces was gradually balanced and that the polarization phenomenon was gradually weakened or disappeared.

**Figure 2:**
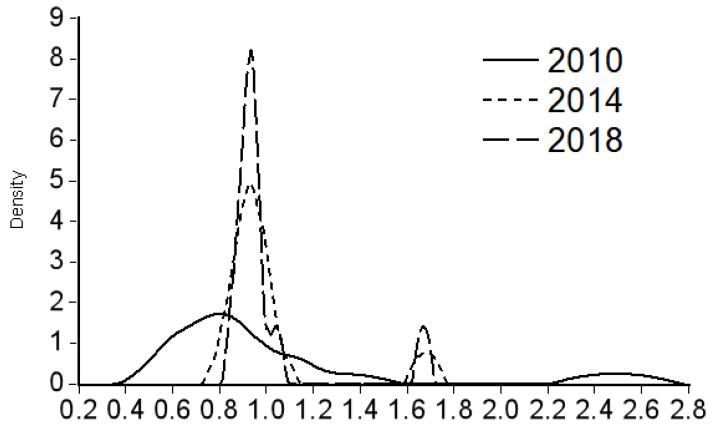
Kernel density estimation of the number of health personnel per 10,000 people in the central economic belt provinces.

The distribution dynamic diagram of provinces in the central economic belt (see Figure 2) shows the following characteristics: First, the Kernel density curve center moved to the right obviously after 2010, and then, the position remained stable, indicating that medical resource investment in provinces in the central economic belt increased first and remained stable after 2010. Second, Kernel density curve center moved to the right obviously after 2010. During the sample investigation period, the height of the main crest of Kernel density curve increased significantly, the width of the main crest decreased significantly, and the Kernel density curve shortened at the right end, indicating that the concentration degree of medical resources in the high input stage in the central economic belt provinces increased, and the range showed a tendency of decreasing. Thirdly, the Kernel density curve gradually evolved into an obvious bimorphous shape and tends to evolve into multi-peaks, indicating that the allocation of medical resources in the central economic belt provinces is unbalanced, with an obvious polarization phenomenon, and presents a trend of multipolar differentiation.

**Figure 3:**
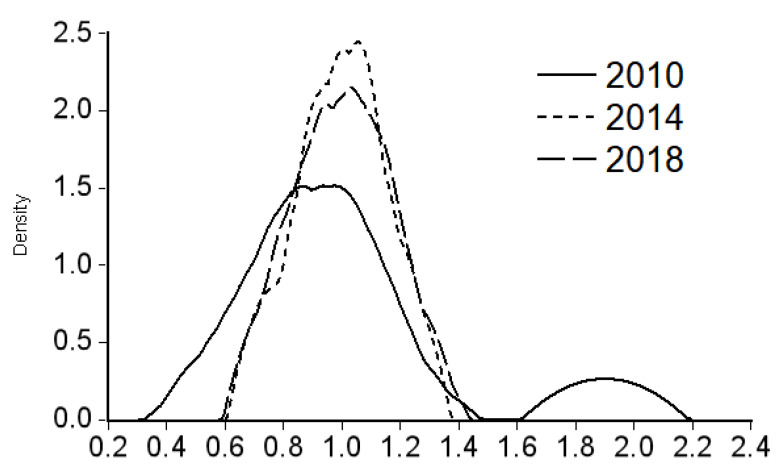
Kernel density estimation of the number of health personnel per 10,000 people in the eastern economic belt provinces.

The distribution dynamic diagram of provinces in the western economic belt (see Figure 3) shows the following characteristics: First, during the sample investigation period, the Kernel density curve center first moved to the right and then remained stable, indicating that, relative to the overall level, the amount of medical resources in the eastern economic belt first increased and then remained stable. Second, the Kernel density curve has a shortened right tail, and the height of the main crest increases while the width decreases, indicating that the concentration of medical resources increases but the range decreases. Thirdly, the Kernel density curve evolved from a bimorphous form to a single peak form with weak multi-peak trend in the high input segment, indicating that the allocation of medical resources in western economic belt provinces is gradually balanced and the polarization phenomenon is gradually weakened, but there is still a trend of multi-pole differentiation.

### 4.2. Data Envelopment Analysis

In this paper, the allocation of medical resources in various provinces and regions of China in 2019 is selected as the research object, and the medical performance of different regions is obtained by using DEAP2.1 software to illustrate the differences in the allocation of medical resources. The results of the DEA evaluation model include comprehensive efficiency (CRSTE), technical efficiency (VRSTE), scale efficiency (SCALE), and return on scale (R-S), as well as redundancy of each input index and deficiency of the output index.

Table 2 shows that the allocation quality of medical resources in China in 2019 is not high and that the differences between regions are significant. Table 3 shows the values of relaxation variables of medical resources in various regions, which can intuitively show the input redundancy and output insufficiency of each province.

From the perspective of comprehensive efficiency, the average comprehensive efficiency of medical and health resource allocation in 31 provinces and cities is 0.580, which is not high overall. Among them, the comprehensive efficiency of medical and health resource allocation in Shanghai, Henan, Guangdong, and Xinjiang is 1.000 (technical efficiency and scale efficiency are also 1.000), and the values of the input indicator relaxation variable and output indicator relaxation variable are both 0, indicating that DEA is effective. The remaining 27 provinces and regions are non-DEA effective (including weak DEA effective and DEA invalid). According to the regional distribution, there is one province in the east (9% of the 11 regions in the east), two in the central region (18% of the 11 regions in the central region), and one in the western region (11% of the 9 regions in the west). It is particularly noteworthy that Ningxia (0.074), Xizang (0.112), and Qinghai (0.169) were ranked as the last three provinces in terms of comprehensive efficiency scores, all of which are in the western region. Thus, it can be seen that the medical resource allocation efficiency of China’s 31 provinces and regions is DEA effective only in the eastern and central regions, while non-DEA effective provinces and regions are mainly distributed in the western regions and generally show differentiation characteristics of higher eastern regions and lower western regions.

From the perspective of technical efficiency, the average technical efficiency of medical resource allocation in 31 provinces and regions in China is 0.702, with a high overall technical efficiency. Among them, there are 8 provinces and regions with technical efficiency = 1.000, all of which are effective in technical efficiency, including not only the four provinces with DEA but also Jiangsu, Zhejiang, Sichuan, and Xizang. The technical efficiency of the remaining 23 provinces and regions is less than 1.000, which is considered as nontechnical efficiency. Among them, Ningxia (0.145), Qinghai (0.220), and Hainan (0.236) have the lowest technical efficiency values, which are all provinces and regions in the central and western regions, among which 2 are in the western region. This indicates that, in 2019, the 23 provinces with nontechnical efficiency and effectiveness did not fully utilized the existing technologies in medical services and that the output is insufficient in the case of a given input.

In terms of scale efficiency, the average scale efficiency of medical resource allocation in 31 provinces and regions in China is 0.809, with a high overall scale efficiency. Among them, the scale efficiency of the four provinces and regions with DEA is 1.000, showing that scale efficiency is effective, while the remaining 27 provinces and regions are all non-scale efficient. Among them, Xizang (0.112), Ningxia (0.511), and Chongqing (0.616) scored the lowest successively, all of which were provinces in the western region. This shows that, in 2019, most provincial health institutions did not operate at the most appropriate investment scale.

According to the status of return to scale, provinces can be divided into three types: diminishing return to scale, constant return to scale, and increasing return to scale. As can be seen from Table 2, at present, only Beijing, Hebei, Tianjin, and Xizang have demands for expanding scale while Shanghai, Henan, Guangdong, and Xinjiang have constant returns to scale. The rest of the provinces and regions are in the stage of diminishing returns to scale, that is, a higher proportion of outputs will be increased if the input is reduced appropriately.

According to the data analysis results, it is not difficult to see that China’s current allocation and balance of medical resources has not reached an ideal state. In terms of the efficiency of medical resource allocation, the eastern region is obviously better, followed by the central region, and the western region is the lowest, presenting a differentiated feature of high east and low west.

## 5. Coupling Analysis of Blockchain and Medical Resource Sharing Mechanism

### 5.1. Application Principal Coupling

**Figure 4:**
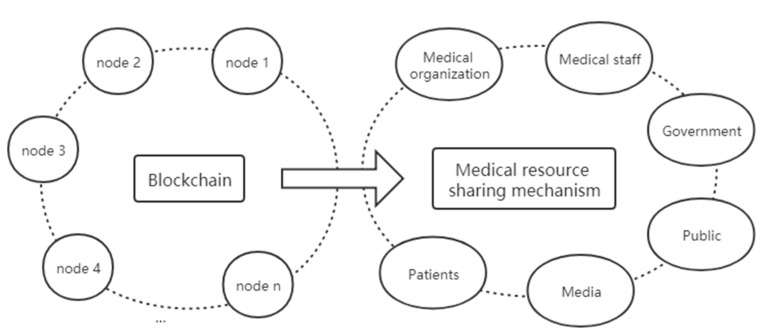
Application principal coupling analysis.

The decentralization and collective maintenance of blockchain technology are reflected in the fact that data in the blockchain technology system is jointly maintained by all participating nodes rather than relying on a traditional central mechanism. The medical resource sharing mechanism is a diversified and multi-level resource sharing mode composed of multiple medical institutions, medical and health practitioners, patients, regulators, and other multiple subjects. Blockchain technology and the medical resource sharing mechanism are based on decentralization and multi-agent collaboration, and each node stores and shares information in accordance with the principle of equality. Therefore, blockchain technology and the medical resource sharing mechanism couple application subjects. The application principal coupling analysis is shown in Figure 4.

### 5.2. Transaction Mechanism Coupling

**Figure 5:**
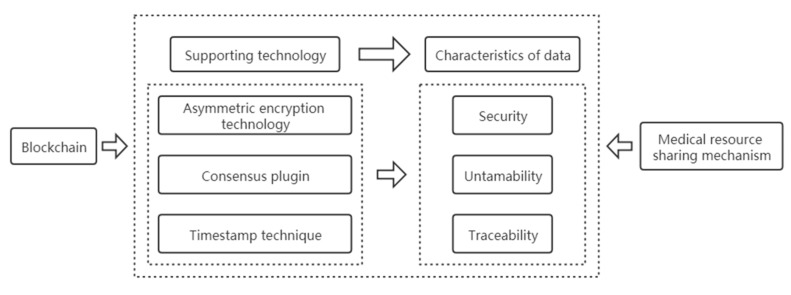
Transaction mechanism coupling analysis.

Blockchain technology transforms the previous “trusters” into “trust machines”. Transactions realized by applying technical principles come from the common trust in trading subjects with the consensus mechanism. At the same time, the timestamp technology adopted by blockchain adds a time dimension to the data on the chain, making it easy to monitor and track and making the data traceable. Medical resource sharing involves medical institutions, medical staff and patients with huge data, blockchain technology adopted by the asymmetric encryption technology, consensus algorithm, and timestamp data security technology; does not tamper with the sex and traceability, real data record, and transfer resources to a great extent; and changes the present medical resource distribution and difficult-to-realize sharing of the status quo. Therefore, the blockchain technology and the medical resource sharing mechanism are coupled in the transaction mechanism. The coupling analysis of transaction mechanism is shown in Figure 5.

### 5.3. Information Flow Level Coupling

The proposal and formation of medical resource sharing mechanism mainly relies on the Internet information technology represented by the blockchain, and the operation of the blockchain involves different levels of computer network system. Therefore, it is possible to compare and analyze the information flow levels of the blockchain technology and medical resource sharing mechanism according to the hierarchical model of information flow in the network.

**Figure 6:**
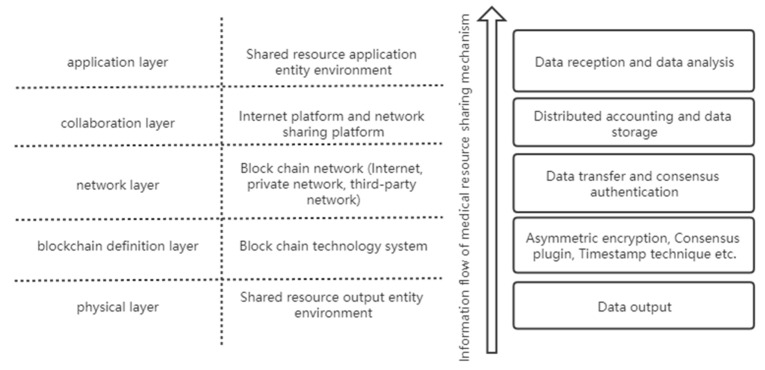
Information flow model.

Figure 6 shows the level of information flow in the medical resource sharing mechanism. The direction of the arrows indicates the direction of information flow transmission and processing. The first layer is the physical layer, indicating the physical environment of medical resource output, which is the starting point in the process of medical resource sharing. The second layer is the blockchain definition layer, which is the definition of a blockchain system in the sharing mechanism. It mainly contains information such as block structure, consensus mechanism, hash algorithm principle, definition of public and private keys, and node authentication method for block, etc. It is an essential level for applying blockchain to a medical resource sharing mechanism. The third layer is the network layer, which is the transmission mode of data and information in the network and the network on which the medical resource sharing mechanism based on the blockchain technology operates. The fourth layer is the collaboration layer, which mainly carries out storage and management of the data generated by the sharing mechanism. A large number of external data involved in the medical resource sharing mechanism will be stored and managed through the Internet platform. The fifth layer is the application layer, which refers to the treatment of acquired resources by the receiving policy of shared resources, including confirmation, analysis, and information feedback.

## 6. Construction of the Medical Resource Sharing Mechanism

In an emergency of special circumstances, the quantity and quality of medical resource differences to a large extent restrict the timely supply and reasonable allocation of medical supplies. During major outbreaks of public health emergencies, no provinces and cities are independently responsible for medical treatment, when large numbers of donated medical supplies, epidemic-prevention materials, and social life supplies are concentrated in one region. The default system makes it difficult to achieve an ideal state of operation and management mechanism; the result is the accumulation of a large number of materials and more obvious contradiction between the supply and demand of medical supplies, which reflects the urgent need for coordination and optimization of the medical resource supply chain. As an important part of information technology system, blockchain technology is highly transparent in its process and highly secure for data. It has made remarkable achievements in many fields of application. At present, the technological development of blockchain has entered the second stage, namely the programmable finance stage of blockchain 2.0, which is developing towards blockchain 3.0. The core of blockchain 3.0 is the application of blockchain technology to decentralize the allocation of global resources and to further extend beyond the fields of currency, economy, and market to build a “programmable society”. With continuous development of the application of blockchain technology, it will also become a strong support for the medical resource sharing mechanism, will greatly improve the degree of medical resource sharing, will narrow the differences in resource allocation between regions, and will realize fairness in medical treatment and the interconnection of medical resources on a larger scale.

**Figure 7:**
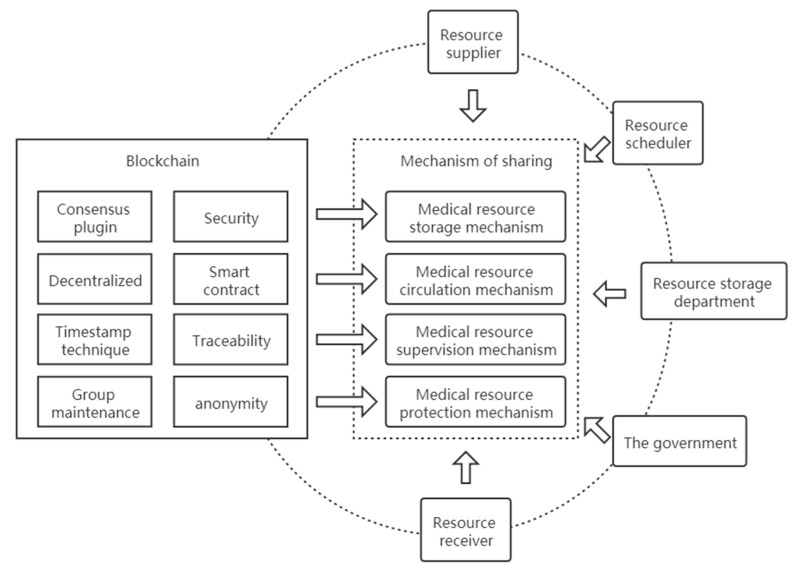
Medical resource sharing mechanism based on blockchain technology.

The characteristics of blockchain technology can promote sharing of medical resources from the four aspects of data storage, circulation, supervision, and protection. Improving the allocation efficiency of medical resources is of great significance for improving the imbalance of resource allocation. Based on the coupling between blockchain and the medical resource sharing mechanism, the medical resource sharing mechanism under the condition of blockchain technology, as shown in Figure 7, consists of four parts:

(1) Medical resource storage mechanism. Based on the consensus mechanism, blockchain technology enables data and information to be stored on the chain in an untamperable manner, effectively preventing information loss or malicious tampering and building a secure, trusted, and untamperable medical resource information library on the basis of the security and reliability of blockchain technology.
(2) Medical resource circulation mechanism. Based on the distributed storage structure of blockchain, data circulation can verify and execute value exchanges on the computer network without a central institution. In the process of resource circulation, the point-to-point transaction and intelligent contract between nodes greatly reduce the transaction cost and improves the transaction efficiency. The data in the blockchain system is open to all nodes, and data records can be queried through the exposed interface while ensuring a high degree of information liquidity.
(3) Medical resource supervision mechanism. Based on the timestamp technology, the time dimension is added to data on the chain, which makes the data traceable. It can not only record the time sequence of transactions but also trace data on the chain according to the timestamp, which is convenient for supervision and tracking.
(4) Medical resource protection mechanism. Based on the collective maintenance and anonymity of blockchain technology, the data in the system are jointly maintained by all participating nodes with maintenance functions. The fault of any node will not affect the operation of the whole system, ensuring stable operation of the whole system. There is no need to disclose identities between nodes, and information can be transmitted anonymously to protect user privacy.

## 7. Discussion

This paper, on the basis of exploring the current situation of medical resource allocation in China, mainly explores blockchain technology under the condition of the construction of a medical resource sharing mechanism using Kernel density estimation and the DEA evaluation model to illustrate the differences existing in the current medical treatment resource allocation features in the country. The results show significant differences between medical resource allocation for different areas and obvious polarization. The health performance level is not high as well, and the problems in the scale efficiency of medical resources constrain further development. Therefore, the government needs to play the role of a resource regulator to reduce the differences in the allocation of medical resources and to establish a medical resource sharing mechanism through coordination and optimization of supply chain management to effectively deal with the “crash” caused by a large number of resources in a short period of time in emergencies.

According to the analysis of the DEA model, it can be seen that the investment scale of medical and health resources in China is relatively large but the efficiency is not high, that the barriers of data sharing still exists, and that the data resources related to medicine and health have not been effectively preserved and shared. At present, it is difficult to share data and information between medical institutions and hardware facilities are a huge problem that restricts the development of “blockchain + medical”. There is an obvious “isolated island” phenomenon in medical institutions, not only because hospitals and patients are worried about information security but also because there is no recognized shared medical resource platform between medical institutions. The disunity of information systems, databases, and coding hinders the sharing of medical resources to a great extent. Aiming at these problems, it is urgent to accelerate the construction of a blockchain platform and to promote exploration of the practical application of a “blockchain + medical treatment”.

As an important part of the information technology system, blockchain technology occupies a pivotal position in both the new Internet industry and the traditional entity industry. With the deepening of the application of “Internet +”, 5G and big data are increasingly closely integrated with the real economy. It is necessary to strengthen the construction of hardware facilities on which the blockchain technology depends; to organically combine the blockchain technology with the Internet, the Internet of Things, and big data; and to promote the development and implementation of the sharing economy. However, in the process of the implementation of “blockchain +”, the three magic weapons of the traditional economic era, regulation–legislation, establishment of institutions, and establishment of thresholds, may not be applicable. The possible problems of medical resource sharing based on blockchain technology can be referred to as an Internet medical treatment to some extent. Its industry characteristics, operation mode, and regulatory key points are quite different from traditional medical doctor–patient legal relationships. In the industrial organization structure, there are more levels of legal relations, such as the relationship between data management optimization and transmission as well as the regulatory and service relations between industry management institutions and various subjects. With wide access to the Internet, the popularization of new medical technologies, the improvement of applications of diagnosis and treatment methods on the Internet, and the enhancement of dependence on electronic data, online medical resources will increasingly affect public health and medical safety and will require new solutions for industry management and legal issues.

## 8. Conclusions

The development of blockchain technology in China is still in the initial stage, and there is no unified legislation on the overall norms and development of the industry. There are also many problems in industrial development in reality: ministry commission, planning commission, the market regulator (food and drug supervision and administration department), and lack of blockchain for medical and health care departments for Internet medical global problems, including access and qualification requirements; diagnosis, classification, and standards; prescription and drug specification; quality supervision and responsibility sharing mechanism; payment and settlement system; data protection; and unified perfect policy laws and regulations such as a property management system.

Based on the consideration of network security issues and data information privacy, “blockchain + medical” poses new challenges to the construction of a regulatory system: on the one hand, it is necessary to strengthen data information management, to avoid the emergence of network security issues and information leakage, and to achieve proper management; on the other hand, it is necessary to promote the sharing of data resources, to accelerate the integration of blockchain technology with the real economy, and to make full use of the advantages of decentralization and de-trust of blockchain technology. It is necessary to strengthen management while avoiding excessive management hindering the integration of blockchain technology with the real economy.

## Figures and Tables

**Table 1:** Definition of variables.

Index Type	Index Name	Variable	Unit
Input indicators	Number of medical and health institutions	*X*_1_	per ten thousand persons
	Number of beds in medical and health institutions	*X*_2_	per ten thousand persons
	Number of health technicians	*X*_3_	per ten thousand persons
Output indicators	Total number of visits	*Y*_1_	-
	Total number of discharged patients	*Y*_2_	-

**Table 2:** Input–output efficiency values of medical resources.

Region		Crste	Vrste	Scale	R-S
Eastern	Liaoning Province	0.479	0.6	0.798	drs
	Jilin Province	0.297	0.458	0.649	drs
	Heilongjiang Province	0.41	0.609	0.673	drs
	Shanghai	1	1	1	-
	Jiangsu Province	0.887	1	0.887	drs
	Zhejiang Province	0.947	1	0.947	drs
	Anhui Province	0.7	0.841	0.833	drs
	Fujian Province	0.419	0.497	0.844	drs
	Jiangxi Province	0.558	0.611	0.913	drs
	Shandong Province	0.837	0.905	0.925	drs
Middle	Beijing	0.915	0.919	0.995	irs
	Tianjin	0.611	0.634	0.964	irs
	Hebei Province	0.587	0.59	0.995	irs
	Shanxi Province	0.27	0.311	0.867	drs
	Inner Mongolia Autonomous Region	0.206	0.322	0.639	drs
	Henan Province	1	1	1	-
	Hubei Province	0.629	0.91	0.692	drs
	Hunan Province	0.814	0.845	0.964	drs
	Guangdong Province	1	1	1	-
	Guangxi Zhuang Autonomous Region	0.589	0.787	0.749	drs
	Hainan Province	0.21	0.236	0.889	drs
Western	Chongqing	0.44	0.715	0.616	drs
	Sichuan Province	0.943	1	0.943	drs
	Guizhou Province	0.482	0.677	0.712	drs
	Yunnan Province	0.659	0.94	0.702	drs
	Tibet Autonomous Region	0.112	1	0.112	irs
	Shaanxi Province	0.456	0.595	0.766	drs
	Gansu Province	0.299	0.405	0.737	drs
	Qinghai Province	0.169	0.22	0.77	drs
	Ningxia Hui Autonomous Region	0.074	0.145	0.511	drs
	Xinjiang Uygur Autonomous Region	1	1	1	-
Average		0.580	0.702	0.809	-

“drs” is short for diminishing returns to scale; and “irs” is short for increasing returns to scale.

**Table 3:** Values of medical resource relaxation variables.

Region		X1	X2	X3	Y1	Y2
Eastern	Liaoning Province	0.000	0.000	1.336	1.199	0.000
	Jilin Province	0.000	1.476	1.586	0.796	0.000
	Heilongjiang Province	0.000	1.187	3.169	0.886	0.000
	Shanghai	0.000	0.000	0.000	0.000	0.000
	Jiangsu Province	0.000	0.000	0.000	0.000	0.000
	Zhejiang Province	0.000	0.000	0.000	0.000	0.000
	Anhui Province	0.000	0.000	1.034	0.577	0.000
	Fujian Province	0.000	0.290	0.000	0.139	0.000
	Jiangxi Province	0.000	0.000	1.131	1.426	0.000
	Shandong Province	1.650	0.975	0.000	0.159	0.000
Middle	Beijing	0.000	5.049	0.000	0.000	0.021
	Tianjin	0.000	1.049	0.000	0.000	0.026
	Hebei Province	2.472	0.000	0.000	0.365	0.000
	Shanxi Province	0.000	0.000	0.675	1.324	0.000
	Inner Mongolia Autonomous Region	0.000	1.789	1.550	0.499	0.000
	Henan Province	0.000	0.000	0.000	0.000	0.000
	Hubei Province	0.000	1.079	0.481	1.293	0.000
	Hunan Province	0.000	0.394	0.143	2.395	0.000
	Guangdong Province	0.000	0.000	0.000	0.000	0.000
	Guangxi Zhuang Autonomous Region	0.000	1.850	0.000	1.230	0.000
	Hainan Province	0.000	5.034	2.069	0.000	0.000
Western	Chongqing	0.000	1.887	3.180	0.798	0.000
	Sichuan Province	1.310	0.410	0.910	0.190	0.000
	Guizhou Province	0.000	0.697	1.529	1.145	0.000
	Yunnan Province	0.000	0.874	1.474	0.710	0.000
	Tibet Autonomous Region	0.000	0.000	0.000	0.000	0.000
	Shaanxi Province	0.000	4.191	0.000	1.270	0.000
	Gansu Province	0.000	0.430	1.401	1.073	0.000
	Qinghai Province	0.000	7.631	4.730	0.160	0.000
	Ningxia Hui Autonomous Region	0.000	17.922	18.126	0.000	0.000
	Xinjiang Uygur Autonomous Region	0.000	0.000	0.000	0.000	0.000

## Data Availability

Publicly available datasets were analyzed in this study. This data can be found here: [http://www.stats.gov.cn/tjsj/ndsj/] and [http://www.nhc.gov.cn/].
